# Is It the Appropriate Time to Stop Applying Selenium Enriched Salt in Kashin-Beck Disease Areas in China?

**DOI:** 10.3390/nu7085276

**Published:** 2015-07-28

**Authors:** Yujie Ning, Xi Wang, Sen Wang, Feng Zhang, Lianhe Zhang, Yanxia Lei, Xiong Guo

**Affiliations:** School of Public Health, Health Science Center, Xi’an Jiaotong University, Key Laboratory of Trace Elements and Endemic Diseases, National Health and Family Planning Commission, Xi’an, Shaanxi 710061, China; E-Mails: yujie.ning@sydney.edu.au (Y.N.); wx231115210@stu.xjtu.edu.cn (X.W.); alison@stu.xjtu.edu.cn (S.W.); fzhxjtu@mail.xjtu.edu.cn (F.Z.); zhanglh@mail.xjtu.edu.cn (L.Z.); leiyanx@163.com (Y.L.)

**Keywords:** selenium, Kashin-Beck disease, selenium content, factor, food item

## Abstract

We aimed to identify significant factors of selenium (Se) nutrition of children in Kashin-Beck disease (KBD) endemic areas and non-KBD area in Shaanxi Province for providing evidence of whether it is the time to stop applying Se-enriched salt in KBD areas. A cross-sectional study contained 368 stratified randomly selected children aged 4–14 years was conducted with 24-h retrospective questionnaire based on a pre-investigation. Food and hair samples were collected and had Se contents determined with hydride generation atomic fluorescence spectrometry. Average hair Se content of 349.0 ± 60.2 ng/g in KBD-endemic counties was significantly lower than 374.1 ± 47.0 ng/g in non-KBD counties. It was significantly higher in the male children (365.2 ± 52.3 ng/g) than in the female (345.0 ± 62.2 ng/g, *p* = 0.002) and significantly higher in the 4.0–6.9 years group (375.2 ± 58.9 ng/g) than the 7.0–14.0 years group (347.0 ± 56.1 ng/g, *p <* 0.01). Gender, living area, Se intake without supplements, Se-enriched salt, oil source and protein intake were identified as significant factors of hair Se contents. Cereals, meat and milk were commonly included as significant food categories that mainly contributed to Se intake without supplement of the whole population. Balanced dietary structure without Se supplement could effectively enhance and maintain children’s Se nutrition. It may be the time to stop applying Se-enriched salt in KBD areas in Shaanxi Province.

## 1. Introduction

Until 1957, scientists have discovered the essential positive effects of Se on animals breaking the limitation of only studying its biologically toxic effects [1,2]. Since then, disorders related to Se-deficiency have been revealed in human beings, mice and other mammalian animals such as pigs and horses [3,4,5,6,7,8,9].

Kashin-Beck disease (KBD) is a serious kind of endemically deformed osteoarthropathy with unclear etiology and pathology. It mainly is distributed from the northeast to southwest China, where the environment is Se-deficient [10]. For decades of research, Se deficiency was found to be one of the main risk factors of KBD [11,12]. As KBD attacks local residents in childhood and clinically manifests [13] in adulthood, effective prevention is the key to protect the health of children as always. Because of Se deficiency being considered a risk factor of KBD, various approaches of supplementation [14], especially Se-enriched salt (sodium selenite: table salt = 1:60,000) [15,16], which is the most economical way for low-income families, had been continuously done in endemic areas. The Se enriched salt turned out to be effective to decrease the incidence of KBD and alleviate the symptoms [11,17]. However, it was suggested to cease the supplement in Shaanxi Province because there are rare new patients in endemic areas without supplements in recent years. But as far as we know, there is no reasonable evidence to support this suggestion.

Nonetheless, apart from Se-enriched salt, some other dietary and/or non-dietary factors may also play positive roles in controlling KBD, according to our recent findings that are based on an analysis of a three-year cohort study [18]. To provide proper evidence for whether it is the time to stop Se supplements in the KBD area in Shaanxi Province, we conducted a sectional investigation based on a prior study to identify which factors are associated with the Se nutrition status in children, in another words, contributing to the controlling of KBD.

## 2. Experimental Section

### 2.1. Study Design and Subjects

**Figure 1:**
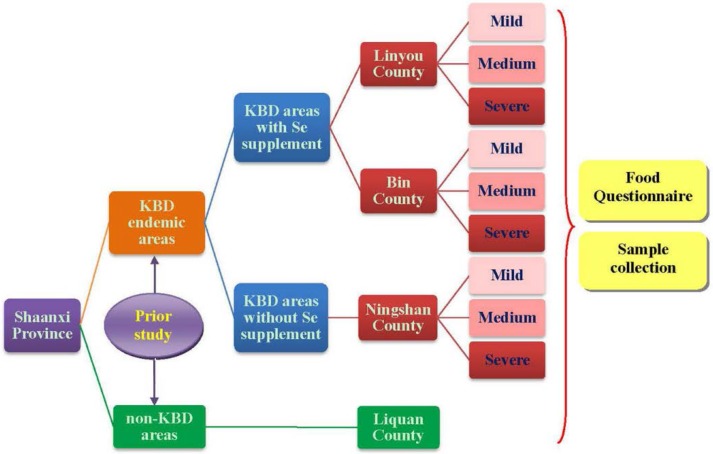
This diagram shows the study design including the stratified random sampling process and the contents of the investigation.

According to the results of prior investigation, we adjusted the contents of questionnaires, most of which were about the food items, such as deleting the unusual foods and adding the ones that the residents often consume. Then, we conducted a 2:1:1 matched cross-sectional investigation in the same counties in Shaanxi Province where the prior investigation was performed from June to September in 2012 and 2013, avoiding summer vacations (study design is shown in Figure 1).

A stratified random sampling method was applied to recruit subjects. According to the design, 2:1:1 matching means that two children in the Se-supplemented KBD area (Linyou and Bin County) were selected *versus* one child in the non-supplemented KBD area (Ningshan County, internal control) and *versus* one child in the non-KBD area (Liquan County, external control) separately. All the subjects were aged 4–14 years and Han nationals, which eliminated the interferences of genetic background and racial bias. The diagnosis criterion of KBD [WS/T 207-2010] in China was used to distinguish patients and healthy children. Subjects with any other osteoarthropathy would be excluded. Based on the results of the pre-investigation, sample size was calculated according to Equation (1).

(1) $$n=\frac{\psi_{\alpha ,\beta \left(\nu_{1},\nu_{2}\right)}^{2}\sum^{\text{​}}S_{i}^{2}/k}{\sum^{\text{​}}\left({\bar{X}}_{i}-\bar{X}\right)/\left(k-1\right)}$$

*k* is the county number, $\sum^{\text{​}}S_{i}^{2}$ is the sum of variances of hair Se content among groups in pre-investigation, $\sum^{\text{​}}{\left({\bar{X}}_{i}-\bar{X}\right)}^{2}$ is the sum of deviation from averages of hair Se contents among groups in pre-investigation, *Α* = 0.05, β = 0.10, $\;\sum^{\text{​}}S_{i}^{2}/k=$ 4258.6, $\;\sum^{\text{​}}\left({\bar{X}}_{i}-\bar{X}\right)/\left(k-1\right)=$ 256.5, $\;\frac{\sum^{\text{​}}S_{i}^{2}/k}{\sum^{\text{​}}\left({\bar{X}}_{i}-\bar{X}\right)/\left(k-1\right)}=$ 16.6, *ν*_1_ = *k*−1,*ν*_2_ = ∞, *k* = 4,$\;\psi_{0.05,0.10\left(3,\infty \right)}=$2.17, *n* = 78.

To ensure the response rate, 15% more subjects than calculated were required, thus sample size increased to 90 per county with 30 subjects in each mild (KBD prevalence < 15%), medium (15% ≤ KBD prevalence < 30%) and severe (KBD prevalence ≥ 30%) endemic area which divided according to the primary prevalence; meanwhile, an equal amount of 90 subjects were required in a non-KBD county. Eventually, 368 eligible subjects were included.

All the subjects and guardians (who were responsible for the children’s meals) were informed and gave their consents about the questionnaire and sample collection. The study was approved by the ethnic committee of Xi’an Jiaotong University (No.2015-070, Date: 2 March 2015).

### 2.2. Data Collection

#### 2.2.1. Anthropometric Measurements

Height and weight measurements were carried out in children in light clothing and without shoes with traditional scales for medical use (RGZ-120, range 60 cm–200 cm/ 5 Kg–120 Kg with 0.1 cm/0.1 Kg measurement error respectively). All results were recorded by a mean value which calculated from two times of measurements, except for a median which would be used instead if a third measurement was taken when the difference between the former two measurements was more than 10%. Then Children’s BMIs were calculated according to Equation (2).

(2) $$BMI=\frac{Weight\left(Kg\right)}{Height^{2}\left(m^{2}\right)}$$

#### 2.2.2. Questionnaire Investigation

Semi-quantitative questionnaires with a series of dietary and non-dietary items (briefly presented in Table 1) were given to and completed by the subjects and their guardians after instructions explained thoroughly by two research assistants who were well-trained and fluent in the local language. The investigation was conducted in house hold except for Ningshan County which was performed in school. The local health and education departments helped a lot in convening subjects and collecting samples.

**Figure 2:**
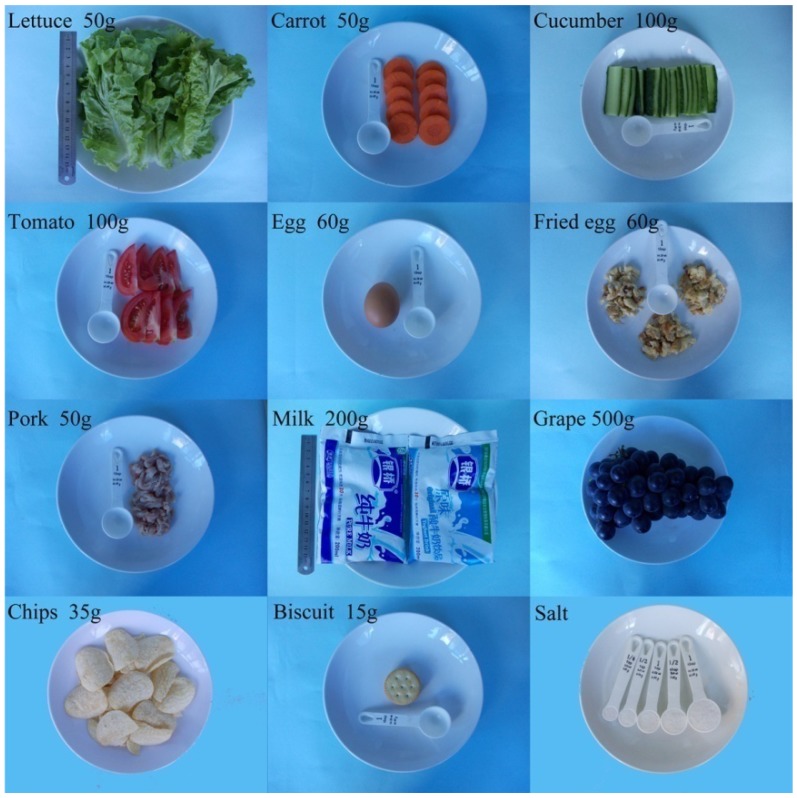
Reference pictures of a small part of food items with household measuring tools in this study.

A 24-h retrospective method was applied to collect the information about food intake. Questionnaires were first given to the subjects and guardians for a browse and administrators asked the children if there were any questions about them. After explanation, inquiry began. Individual dietary information was collected involving food type and intake frequency, and actual consumption of that food per meal in three days which including two working days and one day on the weekend. As most subjects from Ningshan County ate the same dishes for lunch and some of them also had dinner at school, we obtained the menu provided by the school and randomly supervised for three days before the investigation to make sure the dishes were the same as they were described in the menu. Otherwise, we excluded the food items supplied by the school but the students did not eat whilst included the extra consumed foods by the students which were purchased from grocery store or brought from home. Food items consumed at least once every two weeks were listed in the questionnaire, and those which were not on the list were manually recorded. The weight and volume of each consumed food or beverage was estimated using different sizes of household measuring tools such as bowls, cups and spoons and pictures of each regular food in raw and cooked state with reference objects (partly showed in Figure 2) to assure the data as accurate as possible.

The dietary recall of every food item was recorded to calculate the weight in grams as a raw ingredient. When the subjects could not clearly remember the weights of some food items, a median weight which calculated according to the other participants in the same age group with same gender was used. Food intakes were converted into nutrient intakes with the CGDSS 3.0 system that is specially designed for the nutrition analysis of Chinese people. If a food item was not involved in this system, then the food composition table of China, version 2012 was used. The retention factors for food were already taken into consideration based on the traditional Chinese way of cooking.

**Table 1:** Brief contents of questionnaire.

Information Categories	In Details
*Including 17 items of non-dietary items*	
Basic information	Serial number, date, contact number, name, gender, age, nationality, school, address, residence type
Parents	Occupation, education
Birth data	Cesarean, full-term birth or not, ate colostrums or not, feeding methods within six months after birth
Family history of KBD	Number of KBD patients and relationship between them and the children
*Dietary items including the intakes, frequencies and sources*	
Seasonings	3 kinds of table salts, 3 kinds of oils, soy sauce, vinegar and other kinds of seasonings
Grains	Wheat, rice and corn
Vegetables	4 kinds of greens, 4 kinds of cabbage, 6 kinds of tuber vegetables, 6 kinds of cucurbits, 3 kinds of solanaceous vegetables, 5 kinds of root vegetables, 7 kinds of bulb vegetables, 3 kinds of mushrooms, 2 kinds of aquatic vegetables and 2 kinds of flower vegetables
Beans	8 kinds of beans and their products
Egg	Egg white only, yolk only, and whole egg
Meat	3 kinds of livestock meat and products and 2 kinds of haslet, 2 kinds of poultry meat and products and 2 kinds of fish and seafood
Dairy products	4 kinds of dairy products
Nuts	Peanut, sunflower seed, walnut
Fruits	2 kinds of kernel fruit, 5 kinds of stone fruit, 4 kinds of citrus fruit, 3 kinds of melon, 5 kinds of grape and berry, 3 kinds of tropical fruit
Non-staple food	7 kinds of fried-popping food, 16 kinds of confectionery and 4 kinds of soft drinks
Nutritious supplements	Yes or no, if yes then recorded in detail
Taste preferences	Normal, salty, sweat, spicy, sour, greasy
Water sources	Hole water, ditch water, well water, tap water, mineral water
Drinking habit	Unboiled water, boiled water, tea

Frequency categories: never, 1–3 times a month, 1–2 times a week, 3–4 times a week, 5–6 times a week, once a day, 2–3 times a day, more than 3 times a day. Food sources: 1 = grow on their own land, 2 = purchased from market.

#### 2.2.3. Sample Collection and Se Content Determination

With obtained informed consents from all of the subjects and their guardians, occipital hair from 0.5cm to 1.0 cm from the scalp of children were collected by hired barbers whilst the main grain samples were collected by well-trained investigators with the assistance of local related departments. Hair has been being broadly used to estimate the relationship between nutritional elements and human health [19,20,21,22,23,24]. It was firstly washed with neutral detergent solution and then washed in distilled water three times for 2 min followed by being washed in deionized water six times for 2 min to get rid of the shampoo and dirt, *etc.* Se contents of all samples were determined in triplicate by hydride generation atomic fluorescence spectrometry to get the mean values for analyses. All the measurement results were given back to the local related department.

### 2.3. Statistical Analyses

The data were doubly entered and validated by two trained staff members. Statistics for comparison and correlation were analyzed by SPSS18.0. Normality tests were applied before any deeper comparison analyses. Non-parameter methods (e.g., Mann-Whitney U test for pair wise comparisons and Kruskal-Wallis H test for multiple comparisons) were applied when the data was not normally distributed. Pearson’s chi-square test was used to assess the difference in categorical data between the KBD endemic area and non-KBD area. The possible dietary and non-dietary factors thought to be associated with hair Se content and the main food groups contributing to the population’s Se intake without supplement were examined by univariate regression analysis. Only the significant variables with *p* < 0.05 in the univariate regression were included in the multivariate regression analysis to identify a final model contributing to the hair Se level of the population.

## 3. Results

All the measurement results are displayed as mean ± standard deviation (X ± SD).

### 3.1. General Situation

No significant difference of age composition was found among Linyou, Bin and Liquan County (*p* = 0.86) whilst the subjects in each county above were slightly but significantly younger than those in Ningshan County (*p* < 0.01). However, no such difference was found among mild, medium and severe areas within each endemic county (*p >* 0.05). As for the gender ratio, significant differences were found between Linyou and Bin County (*p* = 0.021), Bin and Ningshan County (*p* = 0.013), Ningshan and Liquan County (*p* = 0.035), but no significant difference was analyzed among different areas within each endemic county (*p >* 0.05). The details are displayed in Table 2.

**Table 2:** Description of gender ratio and average age of children in this study.

County	Area	Male (*n*)	Age (years) X ± SD	Female (*n*)	Age (years) X ± SD	Total (*n*)	Age (years) X ± SD
KBD (Se+)							
Linyou	Mild	10	9.3 ± 3.7	20	8.5 ± 3.5	30	8.8 ± 3.5
	Medium	15	8.2 ± 3.1	15	8.7 ± 2.5	30	8.4 ± 2.8
	Severe	15	9.0 ± 3.4	18	7.9 ± 2.9	33	8.4 ± 3.1
	Total	40	8.8 ± 3.3	53	8.4 ± 3.0	93	8.5 ± 3.1
Bin	Mild	23	7.7 ± 3.3	13	9.6 ± 2.7	36	8.4 ± 3.2
	Medium	15	7.8 ± 3.2	17	9.3 ± 2.8	32	8.6 ± 3.1
	Severe	20	8.0 ± 3.2	10	10.1 ± 4.3	30	8.7 ± 3.6
	Total	58	7.8 ± 3.2	40	9.6 ± 3.2	98	8.6 ± 3.3
KBD (Se−)							
Ningshan	Mild	14	10.6 ± 3.1	16	10.6 ± 1.4	30	10.6 ± 2.3
	Medium	9	9.4 ± 2.3	21	10.0 ± 2.7	30	9.8 ± 2.6
	Severe	12	8.8 ± 3.0	15	8.7 ± 2.8	27	8.8 ± 2.9
	Total	35	9.7 ± 2.9	52	9.8 ± 2.5	87	9.8 ± 2.7
Non-KBD							
Liquan	Total	51	8.1 ± 3.2	39	8.7 ± 3.1	90	8.4 ± 3.1
Total		184	8.5 ± 3.2	184	9.1 ± 3.0	368	8.8 ± 3.1

Note: No significant difference of age composition was found among Linyou, Bin and Liquan County with *χ*^2^ = 0.31, *p* = 0.86 whilst that in each county above was significantly different from Ningshan County, *p* < 0.01. Significantly different gender ratio was found between Linyou and Bin County with *χ*^2^ = 5.00, *p* = 0.021, Bin and Ningshan County with *χ*^2^ = 6.19, *p* = 0.013, Ningshan and Liquan County with *χ*^2^ = 4.42, *p* = 0.035, but no significant difference of gender ratio was analyzed among different areas within each endemic county with *p >* 0.05.

Children’s BMIs, calculated according to Equation (2), were 16.8 ± 1.7 for Ningshan, 15.8 ± 1.9 for Liquan, 15.2 ± 2.0 for Linyou and 14.6 ± 1.7 for Bin in descending order. The difference was significant (*χ*^2^ = 74.98, *p* < 0.01). Within each endemic county, only slight significant differences of Children’s BMIs were found in Ningshan County (17.3 ± 1.7 for mild area, 16.3 ± 2.0 for medium area and 17.0 ± 0.8 for severe area, *χ*^2^ = 6.102, *p* = 0.049).

### 3.2. Comparison of Hair Se Content

**Figure 3:**
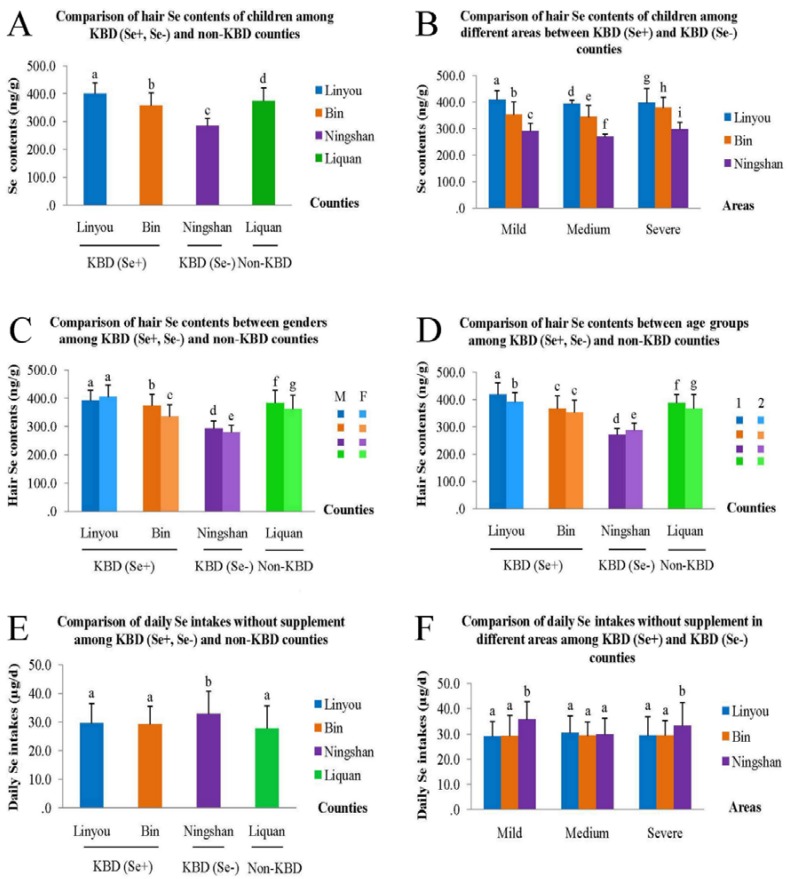
Comparisons of hair Se content and daily Se intake of children in this study. In Figure 3C, M means male, F means female. In Figure 3D, 1 means 4.0–6.9 year age group, 2 means 7.0–14.0 year age group. If the letters above the bars are different, it means that the difference between or among groups was significant, otherwise it means the difference was insignificant.

Average hair Se contents were 399.7 ± 38.8 ng/g in Linyou, 374.1 ± 47.0 ng/g in Liquan, 357.7 ± 45.7 ng/g in Bin and 284.8 ± 26.3 ng/g in Ningshan in descending order and the differences were significant (*χ*^2^ = 202.77, *p <* 0.01, see Figure 3A). Within each endemic county, except for Linyou County, hair Se content was significantly different. In Bin County, it was 378.6 ± 39.9 ng/g in severe areas, 352.6 ± 47.6 ng/g in mild areas and 343.9 ± 42.9 ng/g in medium areas in descending order (*χ*^2^ = 23.31, *p* < 0.01). In Ningshan County, it was 296.8 ± 26.7 ng/g in severe areas, 289.8 ± 30.0 ng/g in mild areas and 269.0 ± 9.4 ng/g in medium areas in descending order (*χ*^2^ = 27.58, *p* < 0.01). The differences are displayed in Figure 3B.

Hair Se content of males (*χ*^2^ = 82.36, *p <* 0.01) and females (*χ*^2^ = 120.45, *p <* 0.01) were significantly different among the four counties. Besides, it was 365.2 ± 52.3 ng/g in all the male children and significantly higher than 345.0 ± 62.2 ng/g in all the female (*Z* = 3.07, *p* = 0.002). Except for Linyou County (392.8 ± 35.0 ng/g for the male and 405.0 ± 40.9 ng/g for the female, *Z* = −0.51, *p* = 0.61), significant differences of hair Se content between the male and the female were found within each of the rest of the counties. In Bin County, 373.7 ± 40.8 ng/g of hair Se content for the male was significantly higher than 334.5 ± 42.8 ng/g for the female (*Z* = 3.82, *p <* 0.01). In Ningshan County, it was 293.0 ± 27.0 ng/g for male and significantly higher than 279.3 ± 24.5 ng/g for female (*Z* = −2.39, *p* = 0.017). In Liquan County, it was 383.3 ± 44.5 ng/g for male and significantly higher than 362.0 ± 48.2 ng/g for female (*Z* = 2.01, *p* = 0.045). More intuitive results of comparisons are shown in Figure 3C.

In each age group, hair Se content was significantly different among the four counties (*χ*^2^ = 53.23, *p <* 0.01 for 4.0–6.9 year group; *χ^2^* = 147.49, *p <* 0.01 for 7.0–14.0 year group. It was 375.2 ± 58.9 ng/g in 4.0–6.9 year group which was significantly higher than 347.0 ± 56.1 ng/g in 7.0–14.0 year group in general (*Z* = −4.56, *p <* 0.01). Except for Bin County (366.2 ± 48.3 ng/g for 4.0–6.9 years old and 353.6 ± 44.2 ng/g for 7.0–14.0 years old, *Z* = −0.97, *p* = 0.33), such differences were found within the rest of the counties. In Linyou County, hair Se content was 418.7 ± 42.0 ng/g for 4.0–6.9 years and significantly higher than 391.2 ± 34.2 ng/g for 7.0–14.0 years (*Z* = −4.22, *p <* 0.01); in Ningshan County, it was 270.3 ± 23.7 ng/g for 4.0–6.9 years and significantly higher than 287.4 ± 26.0 ng/g for 7.0–14.0 years (*Z* = −2.38, *p* = 0.017); in Liquan County, it was 387.4 ± 31.7 ng/g for 4.0–6.9 years old, which was significantly higher than 366.7 ± 52.5 ng/g for 7.0–14.0 years old (*Z* = −4.44, *p <* 0.01), see Figure 3D.

### 3.3. Comparison of Se Content of Food Samples

**Figure 4:**
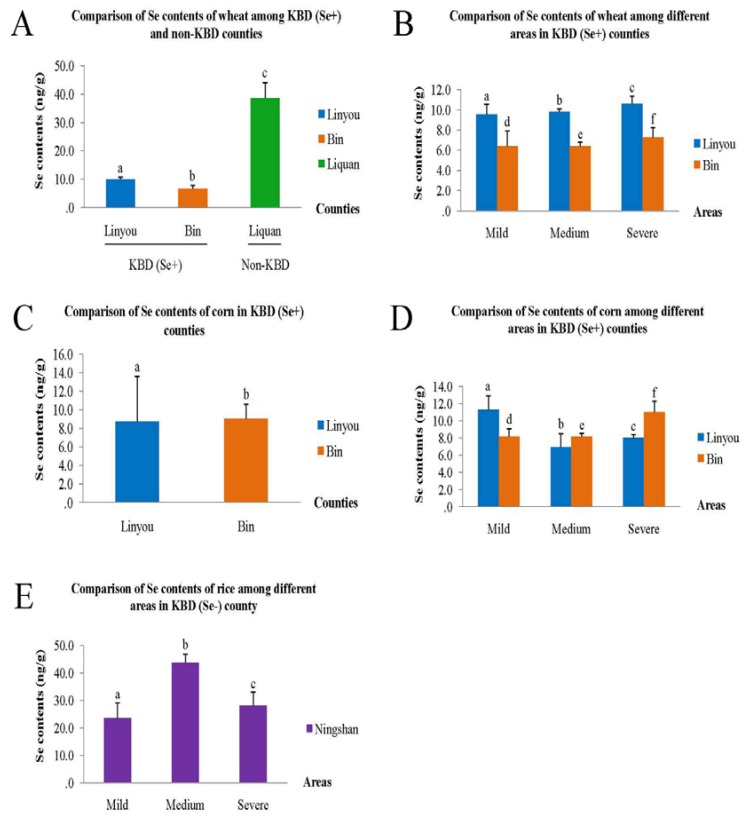
Comparisons of Se content of food samples. If the letters above the bars are different that means the difference between or among groups was significant, otherwise it means the difference was insignificant.

Since wheat is not the main cereal consumed in Ningshan County, the comparisons were just among Linyou, Bin and Liquan County. Generally, wheat Se content was 8.3 ± 2.0 ng/g in endemic counties (10.0 ± 0.9 ng/g in Linyou and 6.7 ± 1.2 ng/g in Bin separately) which was significantly lower than 38.6 ± 5.4 ng/g in Liquan, the non-endemic County (*Z* = −13.68, *p <* 0.01, Figure 4A). Within Linyou and Bin County, it was slightly but significantly increased from mild (9.6 ± 1.0 ng/g, 6.4 ± 1.5 ng/g separately), medium (9.8 ± 0.3 ng/g, 6.4 ± 0.4 ng/g separately) to severe areas (10.6 ± 0.8 ng/g, 7.2 ± 1.0 ng/g separately), see Figure 4B.

Corn is now neither often consumed in Ningshan nor in Liquan and it accounts for only a small part of the staple food in Linyou and Bin County. Corn Se content of 8.7 ± 4.9 ng/g in Linyou was slightly but significantly lower than 9.1 ± 1.6 ng/g in Bin (*Z* = −3.20, *p* = 0.001, Figure 4C). In Linyou County, it significantly decreased from 11.3 ± 1.6 ng/g in the mild area, 8.0 ± 0.4 ng/g in the severe area to 6.9 ± 1.6 ng/g in the medium area (*χ*^2^ = 77.22, *p <* 0.01). In Bin County, it significantly increased from 7.8 ± 0.9 ng/g in the mild area, 8.2 ± 0.4 ng/g in the medium area to 11.0 ± 1.3 ng/g in the severe area (*χ*^2^ = 61.25, *p <* 0.01), see Figure 4D.

Different from the other counties, rice is the main grain consumed in Ningshan County. Rice Se content was 31.9 ± 9.9 ng/g in total and significantly increased from 23.6 ± 5.6 ng/g in the mild area, 28.1 ± 5.0 ng/g in the severe area to 43.7 ± 3.2 ng/g in the medium area (*χ*^2^ = 64.81, *p* < 0.01, Figure 4E).

### 3.4. Relationship between Hair Se Content and Dietary and Non-Dietary Factors

Univariate regression analyses identified six significant non-dietary factors which mainly contributed to hair Se content, *i.e.*, gender, age, BMI, living area, education level of father, and whether they drank colostrums or not and twelve dietary factors, *i.e.*, Se-enriched salt, oil source, type of drinking water, Se intake without supplement, daily energy, protein, carbohydrate, fat, vitamin C, calcium, iron and zinc intakes. Multivariate regression analyses finally included gender, age, living area, Se-enriched salt; oil source, Se intake without supplement, and daily protein intake (see Table 3).

### 3.5. Comparison of Daily Se Intake among Counties and the Contributing Food Categories

In this study, we can tell that without Se-enriched salt, children’s daily Se intake was 29.6 ± 7.0 µg/day in Linyou, 29.3 ± 6.2 µg/day in Bin, 32.9 ± 7.9 µg/day in Ningshan and 27.8 ± 7.9 µg/day in Liquan (*χ*^2^ = 20.24, *p <* 0.01), see figure 3E. There was no significant difference between each of the measurements in Linyou, Bin and Liquan (*p >* 0.05) but all of them were significantly lower than Ningshan (*Z* = −3.52, *p <* 0.01 for Linyou and Ningshan, *Z* = −3.04, *p* = 0.002 for Bin and Ningshan and *Z* = −3.91, *p <* 0.01 for Liquan and Ningshan separately). Among mild, medium and severe areas, it was 29.1 ± 5.9 µg/day, 30.4 ± 8.0 µg/day and 29.4 ± 7.0 µg/day separately in Linyou (*χ*^2^ *=* 0.34, *p* = 0.84), 29.2 ± 6.8 µg/day, 29.3 ± 5.5 µg/day and 29.4 ± 6.4 µg/day separately in Bin (*χ*^2^ *=* 0.01, *p* = 0.99), 35.7 ± 7.4 µg/day, 29.8 ± 5.8 µg/day and 33.2 ± 9.3 µg/day separately in Ningshan (*χ*^2^ = 9.20, *p* = 0.01), see Figure 3F.

Although univariate and multivariate regression analyses identified different food categories that significantly contributed to daily Se intake of children in different areas, cereals (wheat for Linyou, Bin and Liquan County and rice for Ningshan County), meat mainly livestock and poultry, and milk were included in every county by both methods, details are displayed in Table 4).

**Table 3:** Significant influence factors of hair Se content of children identified by univariate and multivariate regression analyses.

Factors	*B*	95.0% CI for *B*		*t*	*P*	*R*
		Lower bond	Upper bond			
Univariate regression						
*Non-dietary factors*						
Gender*	−20.12	−31.90	−8.35	−3.36	0.001	0.17
	(−13.36)	(−22.14)	(−4.58)	(−2.99)	(0.003)	-
Age (year) *	−4.58	−6.46	−2.71	−4.80	<0.01	
	(−2.46)	(−4.04)	(−0.87)	(−3.05)	(0.002)	
BMI	7.25	4.33	10.17	4.88	<0.01	0.25
Living area *	−25.14	−38.80	−11.47	−3.62	<0.01	0.19
	(−89.69)	(−106.01)	(−73.37)	(−10.81)	<0.01	-
Education level of father	28.48	16.32	40.64	4.61	<0.01	0.23
Drank colostrum or not	−41.23	−57.72	−24.74	−4.92	<0.01	0.25
*Dietary factors*						-
Se-enriched salt or not *	−48.43	−59.31	−37.56	−8.76	<0.01	0.42
	(−103.03)	(−120.60)	(−85.46)	(−11.53)	<0.01	-
Oil source *	27.41	15.59	39.24	4.56	<0.01	0.23
	(15.25)	(4.12)	(26.37)	(2.70)	(0.007)	-
Type of drinking Water	58.56	47.17	69.96	10.11	<0.01	0.47
Daily Se intake (without Se-salt) *	2.17	0.37	3.98	4.89	0.004	0.35
	(14.36)	(2.62)	(26.11)	(2.41)	(0.017)	-
Daily energy intake	0.02	0.01	0.04	2.91	0.004	0.15
Daily protein intake *	0.73	0.35	1.11	3.74	<0.01	0.19
	0.79	0.14	1.44	2.39	0.017	-
Daily carbohydrate intake	−0.15	−0.26	−0.04	−2.64	0.009	0.14
Daily fat intake	−1.70	−2.18	−1.22	−6.93	<0.01	0.34
Daily vitamin C intake	0.70	0.43	0.97	5.15	<0.01	0.26
Daily Ca intake	0.08	0.05	0.11	5.84	<0.01	0.29
Daily Fe intake	0.72	0.29	1.16	3.26	0.001	0.17
Daily Zn intake	7.05	5.65	8.46	9.86	<0.01	0.46
Multivariate regression *						
Total	-	-	-	-	-	0.77

Note: In parentheses are the multivariate regression results of the same variable marked by symbol “*”.

**Table 4:** Significant dietary factors of daily Se intakes of children without supplements identified included by multivariate regression analyses.

Factors	KBD (Se+)		KBD (Se−)		Non-KBD		Total	
	*P*	*R*	*P*	*R*	*P*	*R*	*P*	*R*
Wheat *	<0.01	† 0.24	0.08	0.19	<0.01	† 0.48	0.01	0.22
Rice *	0.22	0.09	0.01	† 0.28	0.31	0.11	<0.01	0.24
Greens *	0.04	0.15	0.13	0.16	0.12	0.17	0.01	0.14
Tuber vegetable *	0.01	† 0.23	0.03	0.23	0.06	0.20	<0.01	0.22
Solanaceous *	0.01	† 0.18	0.18	0.14	0.01	0.29	<0.01	0.24
Bulb vegetable *	0.02	† 0.17	0.11	0.17	0.06	0.20	<0.01	0.20
Aquatic vegetable	0.02	† 0.17	0.16	0.15	0.47	0.08	<0.01	0.22
Livestock meat *	0.03	† 0.16	0.04	† 0.22	0.04	† 0.21	<0.01	0.20
Poultry meat *	0.12	0.11	0.50	0.07	0.23	0.13	0.04	0.11
Milk *	<0.01	† 0.18	0.04	† 0.21	0.04	† 0.22	<0.01	0.21
Nuts *	0.16	0.10	0.01	0.27	0.19	0.14	0.01	0.14
Kernel fruit	0.06	0.14	0.18	0.14	0.04	† 0.21	0.75	0.02
Beverage *	0.23	0.09	<0.01	† 0.36	0.25	0.12	<0.01	0.20
Total	<0.01	0.53	<0.01	0.51	<0.01	0.57	<0.01	0.69

Note: Data of *B* and its 95% CI are not shown in this table. These significant dietary factors of daily Se intakes of children without supplements were included by multivariate regression analyses from the significant factors which firstly included by univariate regression analysis with *p <* 0.05. *R* value marked by symbol “†” means the corresponding factor included by multivariate regression analysis was significant for daily Se intake of children in that area. Factor labeled by symbol “*” means the factor included by both univariate and multivariate regression analyses was significant for daily Se intake of the whole population in this study.

## 4. Discussion

KBD could be traced back to the 16th century, and hundreds of years of research have been applied worldwide. It was found to be tightly related to Se-deficiency in the 1970s in China. Environmentally low Se leads to insufficiency *in vivo* via the food chain. Naturally, various methods of Se supplement such as Se-enriched salt, yeast, egg, tea, milk and other food, oral sodium selenite [25], spraying Se on crops and Se-enriched fertilizer [26,27] had been applied to improve the Se nutritional status of local residents living in Se-deficient areas. As expected, supplements have obviously promoted the repair of metaphysis pathological changes of KBD and greatly reduced the incidence [11].

Although Se deficiency was not a direct cause of KBD, it was demonstrated to be a major risk factor. Se supplements remarkably improved the pathological changes of KBD and efficiently contributed to the control of the incidence [11,28]. As time goes by, barely any new cases have been diagnosed nationwide. In Shaanxi Province, there are a few KBD areas without Se supplements, for instance Ningshan County, in which the incidence have also been controlled. This brought up the proposal of ceasing the Se-enriched table salt in other supplemented endemic areas. The reason for the disappearing KBD in non-Se-supplemented areas must be identified before taking action on this proposal.

In this study, hair Se content of children from KBD supplemented counties (Linyou and Bin), KBD non-Se-supplemented county (Ningshan) and non-KBD county (Liquan) were much higher than 200 ng/g which was considered as the critical value of non-endemic level [29]. Gender, age, living area, Se-enriched salt, oil source, Se intake without supplement and daily protein intake were included as the major factors that influenced the Se status of children.

About the non-dietary factors, hair Se level was significantly higher in male than that in female which was similar with S. Letsiou’s finding [30] whilst a contrary result was reported in adults from seleniferous areas in the United States [31]. There was no consensus of the relationship between age and Se nutritional status in human beings. We found it was significantly higher in the 4–6.9 year group than in the 7–14 year group. This decline was also identified between teenagers and middle aged groups [30]; however, it was found that it increased with age in the centenarians [24] whilst Chiristine A Swanson *et al.* [31] and Veronique Ducros *et al.* [32] pointed out that age was not associated with tissue selenium content. As for living areas, it was probably linked with the different concentrations of Se in the environment, especially the arable land [10]. This explained well the importance of the oil source that was either produced in the residents’ Se-deficient land or purchased from KBD free areas. However, hair Se content among mild, medium and severe areas within each KBD County showed no consistent trend whilst it was consistently way higher than the non-endemic level, 200 ng/g.

In spite of Se-enriched salt still playing a role in maintaining the Se nutrition of children, dietary Se intake without supplements also considerably contributes to the Se nutrition of children. It is well known that Se can only be used after being incorporated into proteins and then being further transformed into selenocysteine, the active form of Se [33,34]. Protein is one of the basic gradients of selenoprotein for storage or utilization, we identified the intake of which was positively related to the Se status of children [35,36]. The average of daily Se intake of children in Linyou, Bin County and Liquan County were very close but significantly lower than that in Ningshan County. Cereals, meat and milk were commonly included as significant food categories which contributed to daily Se intake without supplement of the whole population (see Table 4), which is similar to the contributions of food groups to total population in the UK [37]. Only wheat is the main cereal in the former three counties whilst rice is consumed daily in Ningshan County. Accordingly, Se concentration of rice in Ningshan County was slightly lower than that of wheat in Liquan but it was 3–5 folds higher than that of wheat in Linyou County. Besides, in Ningshan County, egg and milk have been strictly supplied in schools every working day since 2009 and the students have been eating the same diversified food provided by the school for a couple of years. These are probably the reasons why the children in Ningshan County had the highest Se intake without supplements.

Disregarding the area division, the food categories majorly contributing to the Se intake without supplement were wheat, rice, livestock and poultry meat, green and solanaceous vegetables, tuber and bulb vegetables, nuts, milk and beverages. Except for the uncertainty of some beverages, Se gained from the rest of the food are mainly in organic form (e.g., selenomethionine and selenocysteine), which has much higher bioavailability and longer half-life than the inorganic form (e.g., selenite and selenate). In addition, the organic form can increase blood Se level more rapidly and to a greater extent than the inorganic form [1,34]. Diet structure in all the counties is getting better and more diversified compared to the last couple of decades when the residents’ tables mainly served ditch water, corn, wheat and potatoes while lacking meat, egg, milk, beans and other vegetables because of the bad economy and inconvenient transportation [38,39,40].

## 5. Conclusions

Although Se-enriched salt still plays a role in keeping children’s Se nutritional status, the better source of food and improved dietary structure may enhance and maintain the children’s Se nutrition in a better and safer way, which indicates that more attention should be paid to the quality and diversity of food for better comprehensive nutrition of children. Is it now the time to stop applying selenium supplementation in KBD endemic areas in Shaanxi Province, China? Based on the pros and cons, the answer may be yes. However, so far, no cohort study longer than three years has been conducted to demonstrate whether ceasing Se-enriched salt in KBD endemic areas would create no risk of a resurgence of KBD. In addition, no clear evidence could estimate how long it takes to get KBD and there are occasionally a few new suspect cases in Qinghai Province and Tibet (data unshown) in China. Therefore, for safety’s sake, we suggest ceasing the supplement from one county for a start while continuing to monitor the Se status of children and KBD incidence twice a year for at least three years to prevent increase in incidence, and then expand the program to the rest of the endemic areas in or even beyond Shaanxi Province.
